# Supplementary material for: Content-rich biological network constructed by mining PubMed abstracts
Source: BMC Bioinformatics. 2004 Oct 8;5:147. doi: 10.1186/1471-2105-5-147 (PMC528731; doi:10.1186/1471-2105-5-147)
Supplement: Additional File 2 — The original results of the above study (non-essential files are deleted to keep the file size under the limit set by BMC bioinformatics). [file 1471-2105-5-147-S2.bz2 › chilibotAdditionalFile2/dip30/153ID1423597E548/html/CDK4_CCND1.html]

 


 **CDK4** and **CCND1** 
  
Found 880 abstracts in PubMed, retrieved 30.  
 

 What does Google say? 
 PDF only 
| .edu only 

---

**Interactive relationship** (e.g. stimulation, inhibition, etc)

**Inhibitory relationship**- Cytoplasmic cyclin D  [ **CCND1** ]  1 assembled with cyclin dependent kinase 4  [ **CDK4** ]  CDK 4  [ **CDK4** ] , and the CDK inhibitors p21Cip1 and p27Kip1.  Ref: 12872751 Kokubyo Gakkai Zasshi, 2003
- Rapamycin and its analogues also prevent cyclin dependent kinase CDK activation, inhibit retinoblastoma protein phosphorylation, and accelerate the turnover of cyclin D1  [ **CCND1** ] , leading to a deficiency of active  **CDK4**  cyclin D1  [ **CCND1** ]  complexes, all of which potentially contribute to the prominent inhibitory effects of rapamycin at the G1 S boundary of the cell cycle.  Ref: 12864941 Clin Breast Cancer, 2003
**Stimulatory relationship**- Increased expression, or stability, of cyclin D1  [ **CCND1** ]  molecules may cause sufficient  **cdk4**  activation to produce retinoblastoma protein phosphorylation independently of mitogenic signals.  Ref: 12834607 J Comp Pathol, 2003
**Neutral relationship**- The three human D type cyclins, cyclin D1  [ **CCND1** ] , D2 and D3 share the ability to bind to and activate  **cdk4**  and 6.  Ref: 12853979 Oncogene, 2003

**Non-interactive relationship** (e.g. studied together, co-existance, homology, etc.)

- In contrast, degradation of the AhR in HepG2 liver cancer cells resulted in decreased G0 G1 > S phase progression, and this was accompanied by decreased expression of cyclin D1  [ **CCND1** ] , cyclin E, cyclin dependent kinase 2 cdk2, and  **cdk4** .  Ref: 12761348 Mol Pharmacol, 2003
- The restored ZD AZ forestomach epithelium displayed strong expression of Bax, a proapoptotic protein, and weak staining of cyclin D1  [ **CCND1** ]  and its catalytic partner  **Cdk4** , key regulatory proteins controlling G 1 to S progression.  Ref: 12873989 Cancer Res, 2003
- To improve understanding of the role of this pathway in spermatogenesis, and its subversion in TGCTs, we examined immunohistochemical expression patterns of  **CDK4** , p16INK4a, p15INK4b, and pRB, and established an in situ assay for cyclin D  [ **CCND1** ]  mediated phosphorylation of serine795, a phosphorylation event critical for neutralization of pRB s growth restraining ability.  Ref: 12754735 J Pathol< MedlineTA>J Pathol, 2003
- Sp1 blocked assembly of cyclin D1  [ **CCND1** ]   **Cdk4**  p21WAF1 Cip1 complex formation whose integrity is critical for G1 > ; S transition.  Ref: 12796485 J Biol Chem< MedlineTA>J Biol Chem, 2003
- Arsenic increased expression of the P21 protein and decreased levels of cyclin A, cyclin B1 and cyclin D1  [ **CCND1** ] , but expression of CDK2,  **CDK4** , CDK6, and cyclin E were not affected.  Ref: 12883267 Pancreas, 2003
- Monensin decreased the levels of CDK2, CDK6, cdc2, cyclin A, cyclin B1, cyclin D1  [ **CCND1** ]  and cyclin E proteins but did not alter  **CDK4**  protein.  Ref: 12792794 Int J Oncol, 2003
- While Tax competes with  **CDK4**  for p16 binding, thus suppressing p16 inhibition of  **CDK4** , Tax also binds to cyclin D  [ **CCND1** ]  s with concomitant increases in both  **CDK4**  activity and the phosphorylation of cyclin D  [ **CCND1** ]  s.  Ref: 12779347 Biochemistry, 2003
- Sp1 Inhibits proliferation and induces apoptosis in vascular smooth muscle cells by repressing p21WAF1 Cip1 transcription and cyclin D1  [ **CCND1** ]   **Cdk4**  p21WAF1 Cip1 complex formation.  Ref: 12796485 J Biol Chem< MedlineTA>J Biol Chem, 2003
- Overall, our results indicate that Rho kinase dependent stress fiber formation is required for sustained activation of the MEK ERK pathway and the mid G 1 phase induction of cyclin D1  [ **CCND1** ] , but not for other aspects of  **cdk4**  or cdk2 activation.  Ref: 12773570 Mol Cell Biol, 2003
- Positive staining for cyclin D1  [ **CCND1** ] , cyclin E, cyclin A, cyclin B1, cdk2,  **cdk4** , cdc2, p53, p21, and p27 was observed in 63%, 66%, 31%, 32%, 51%, 77%, 71%, 43%, 35%, and 60% of the 82 carcinomas, respectively.  Ref: 12792921 Hum Pathol, 2003
- The high rate of cell proliferation was accompanied by overexpression of cell cycle progression and tumorigenesis biomarkers, including proliferating cell nuclear antigen, cyclin D1  [ **CCND1** ] , cyclin dependent kinase 4  [ **CDK4** ] , p53, cytokeratin 14, epidermal growth factor receptor, and by a reduced rate of apoptosis.  Ref: 12874033 Cancer ResCancer Res, 2003
- In fetal cells the induction of apoptosis and differentiation by RA was obtained via inhibition of cyclin D1  [ **CCND1** ]   **cdk4**  activity, as result of an increased binding to the p16 inhibitor.  Ref: 12753235 Liver, 2003
- Quantitative immunochemical evaluation of the expression of certain proteins controlling the cell cycle showed an increase in p27kip1, cyclin D  [ **CCND1** ] , and  **cdk4**  positive cells, with a decrease in cyclin E and cdk2 positive cells.  Ref: 12805303 J Neurosci, 2003
- EGF administration after PH also significantly affected the activity of the cyclin D1  [ **CCND1** ]   **cdk4**  and cyclin E cdk2 complexes, mainly by changing their time progression it accelerated the increase in activity to 18h and caused a subsequent drop in activity after 34h.  Ref: 12876382 Cell Physiol Biochem, 2003
- for cyclin D1  [ **CCND1** ] , cyclin A,  **cdk4** , p21, and p53 was more frequent in higher grade tumors.  Ref: 12792921 Hum Pathol, 2003
- Western blotting analysis revealed that the protein levels of cyclin A, cyclin E, and cyclin dependent kinase CDK 2 but not cyclin D1  [ **CCND1** ]  and  **CDK4**  decreased after progesterone treatment, but those of CDK inhibitory proteins, p21 and p27, increased.  Ref: 12810531 Endocrinology< MedlineTA>Endocrinology, 2003
- Novel, potent and selective cyclin D1  [ **CCND1** ]   **CDK4**  inhibitors indolo a pyrrolo c carbazoles.  Ref: 12824014 Bioorg Med Chem Lett, 2003
- In relation to these effects, Tax has been shown to interact with two components of the INK4  **CDK4**  6 Rb pathway, p16 and cyclin D  [ **CCND1** ]  s.  Ref: 12779347 Biochemistry, 2003
- These effects were highly specific, as the kinetics of cyclin D  [ **CCND1** ]   **cdk4**  activation was unaltered.  Ref: 12842876 J Biol Chem< MedlineTA>J Biol Chem, 2003
- On the other hand, EPA inhibited cyclin dependent kinase 4  [ **CDK4** ]   **CDK4**  activation and cyclin D1  [ **CCND1** ]  protein induction, a critical step for G1 S progression.  Ref: 12811822 J Cell Physiol, 2003
- Moreover, Rb phosphorylation, which lies immediately downstream of the cyclin D1  [ **CCND1** ]   **Cdk4**  p21WAF1 Cip1 complex, was blocked either by Sp1 overexpression or antisense p21WAF1 Cip1.  Ref: 12796485 J Biol Chem< MedlineTA>J Biol Chem, 2003
- In a series of 23 surgical biopsies of grade II and III oligodendroglioma, cyclin D1  [ **CCND1** ] , E, A, B1,  **CDK4**  6, CDK2, Cdc2 and p27 Kip.1 have been studied by immunohistochemistry and Western blot.  Ref: 12873740 Neurosci Lett, 2003
- Expression of cyclin D1  [ **CCND1** ]  and cyclin dependent kinase 4  [ **CDK4** ]  was also examined with double label immunohistochemistry.  Ref: 12754083 Brain Res Bull, 2003
- Inhibition of either MLCK or Rho kinase blocked sustained ERK signaling, but only Rho kinase inhibition allowed for the induction of cyclin D1  [ **CCND1** ]  and activation of  **cdk4**  via Rac Cdc42.  Ref: 12773570 Mol Cell Biol, 2003
- No changes were observed in the expression levels of the G1 regulatory molecules cyclin D1  [ **CCND1** ]  and  **cdk4** , but cell cycle arrest and apoptosis induction was accompanied by accumulation of the cdk inhibitor p21 in both cell lines.  Ref: 12846895 Br J Haematol, 2003
- Binding of these CKI s to both the cyclin D1  [ **CCND1** ]  CDK 4  [ **CDK4** ]  complex and the cyclin E CDK 2 complex were increased and the kinase activities of these complexes were reduced with increasing dietary corticosterone.  Ref: 12807724 Carcinogenesis< MedlineTA>Carcinogenesis, 2003
- Furthermore, induction of cyclin D  [ **CCND1** ]  and cyclin dependent kinase 4  [ **CDK4** ]  mRNA and protein was blocked upon inhibition of caspase 6, but not caspase 3.  Ref: 12794135 J Immunol, 2003
- Levels of cyclin D1  [ **CCND1** ] , cyclin E, CDK 2 and CDK 4  [ **CDK4** ]  were reduced in carcinomas from corticosterone treated rats.  Ref: 12807724 Carcinogenesis< MedlineTA>Carcinogenesis, 2003
- These inhibitors also effect strong G1 arrest in these cell lines and inhibit Rb phosphorylation at Ser780 consistent with inhibition of cyclin D1  [ **CCND1** ]   **CDK4** .  Ref: 12824014 Bioorg Med Chem Lett, 2003
- In contrast, proliferative ZD Wt forestomach showed strong expression of Bcl  [ **CCND1** ]  2, an antiapoptotic protein, and overexpression of cyclin D1  [ **CCND1** ]   **Cdk4** .  Ref: 12873989 Cancer Res, 2003
- In the lactacystin treated OLGcs, there was a dose dependent decrease in the number of cells incorporating bromodeoxyuridine and in the activity of the complexes cyclin D  [ **CCND1** ]   **cdk4**  and cyclin E cdk2.  Ref: 12805303 J Neurosci, 2003
- High correlation was found between cyclin A and p53 expression, between cyclin D1  [ **CCND1** ]  and  **cdk4**  expression, between  **cdk4**  and Ki 67 expression, and between p21 and Ki 67 expression.  Ref: 12792921 Hum Pathol, 2003
- Among these factors, positive staining for cyclin D1  [ **CCND1** ] ,  **cdk4** , and p53 was significantly frequent in advanced stage tumors,.  Ref: 12792921 Hum Pathol, 2003
- Cyclin D1  [ **CCND1** ]  and cyclin dependent kinase 4  [ **CDK4** ]  immunoreactivity appeared in these glial cells in parallel with the expression of PCNA.  Ref: 12754083 Brain Res Bull, 2003
- However, the activity of cyclin E CDK2 was decreased in a concentration and time dependent manner and the activity of cyclin D  [ **CCND1** ]   **CDK4**  was inhibited when serum starved synchronized cells were released from synchronization.  Ref: 12781329 Biochem Pharmacol, 2003
- Overexpression of cyclin D1  [ **CCND1** ] , the regulatory subunit of cyclin dependent kinases  **cdk4**  and cdk6 involved in cell cycle control, has often been found in breast cancer and other types of human cancer.  Ref: 12834607 J Comp Pathol, 2003
- These results suggest that the suppression of cell cycle progression in the G1 phase by 5GG was mediated in MCF 7 cells, at least in part, by either the inhibition of cyclin D  [ **CCND1** ]   **CDK4**  and cyclin E CDK2 activity or the induction of the CDK inhibitors p27 Kip and p21 Cip.  Ref: 12781329 Biochem Pharmacol, 2003
- Genetic characterization was performed using cytogenetics, fluorescence in situ hybridization, flow cytometry, and molecular techniques for analysis of the genes implicated in cell cycle control, amplification of MDM2,  **CDK4** , and Cyclin D1  [ **CCND1** ] , and mutations in the p53 gene.  Ref: 12808123 Lab Invest, 2003
- In our study, we screened HCCs resulting from HCV infection 51 cases, HBV infection 26 cases or excess alcohol intake 23 cases for alterations in genes involved in the RB1 pathway p16 INK4a, p15 INK4b, RB1,  **CDK4**  and cyclin D1  [ **CCND1** ] , the p53 pathway p53, p14 ARF and MDM2 and the Wnt pathway beta catenin, APC.  Ref: 12845670 Int J Cancer, 2003
